# Scientific productivity: An exploratory study of metrics and incentives

**DOI:** 10.1371/journal.pone.0195321

**Published:** 2018-04-03

**Authors:** Mark D. Lindner, Karina D. Torralba, Nasim A. Khan

**Affiliations:** 1 Center for Scientific Review, National Institutes of Health, Bethesda, Maryland, United States of America; 2 Division of Rheumatology, Loma Linda University, Loma Linda, California, United States of America; 3 Division of Rheumatology, Department of Internal Medicine, University of Arkansas for Medical Sciences, Arkansas, United States of America; 4 Central Arkansas Veterans Healthcare System, Little Rock, Arkansas, United States of America; CPERI, GREECE

## Abstract

Competitive pressure to maximize the current bibliometric measures of productivity is jeopardizing the integrity of the scientific literature. Efforts are underway to address the ‘reproducibility crisis’ by encouraging the use of more rigorous, confirmatory methods. However, as long as productivity continues to be defined by the number of discoveries scientists publish, the impact factor of the journals they publish in and the number of times their papers are cited, they will be reluctant to accept high quality methods and consistently conduct and publish confirmatory/replication studies. This exploratory study examined a sample of rigorous Phase II-IV clinical trials, including unpublished studies, to determine if more appropriate metrics and incentives can be developed. The results suggest that rigorous procedures will help reduce false positives, but to the extent that higher quality methods are accepted as the standard of practice, the current bibliometric incentives will discourage innovative studies and encourage scientists to shift their research to less informative studies of subjects that are already being more actively investigated. However, the results also suggest that it is possible to develop a more appropriate system of rewards. In contrast to the current bibliometric incentives, evaluations of the quality of the methods and reproducibility of the results, innovation and diversity of thought, and amount of information produced may serve as measures and incentives that maintain the integrity of the scientific literature and maximize scientific progress.

## Introduction

The value of science is that it can address important questions about the real world. Ideally, rigorous studies are designed to provide the most critical challenge that can be devised for each new hypothesis. Hypotheses that pass the critical initial test are then confirmed and validated by demonstrating that they can be reliably reproduced [1–4]. While this process is effective at increasing knowledge, it is dependent on the production and publication of valid results regardless of the outcome (i.e., whether the results are positive, statistically significant; or negative, nonstatistically significant). As Chalmers et al. have stated, “Good research ideas often do not yield the anticipated results…[but] these disappointments should not be deemed wasteful; they are simply an inevitable feature of the way science works” [5].

**Fig 1:**
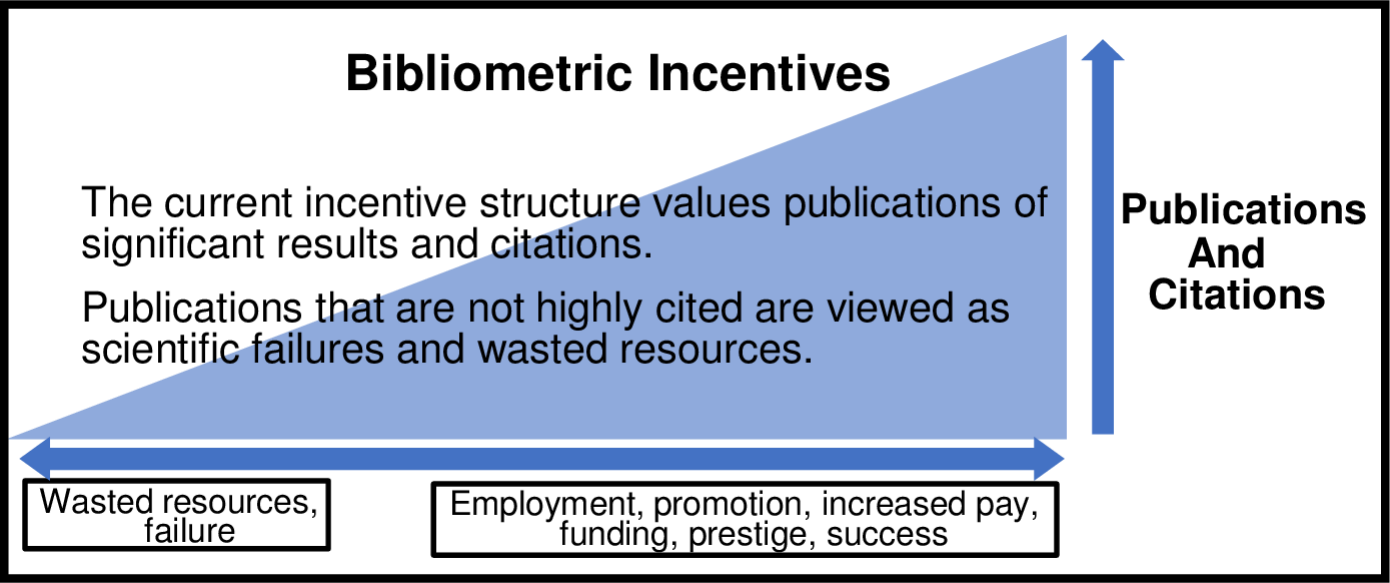
The current bibliometrics incentives model.

Unfortunately, the current system of incentives does not adequately support this normative ideal. There is little reward for ensuring that new findings are valid or for conducting critical studies to filter out results that cannot be reliably reproduced. Instead, there is tremendous competitive pressure to claim primacy of discovery and to maximize the numbers of publications and citations [6]. Only one out of every eight new PhDs survive in their careers to become principal investigators [7], and their success is based on who can publish the most papers and garner the most citations, especially in journals with high impact factors [8] (Fig 1).

**Fig 2:**
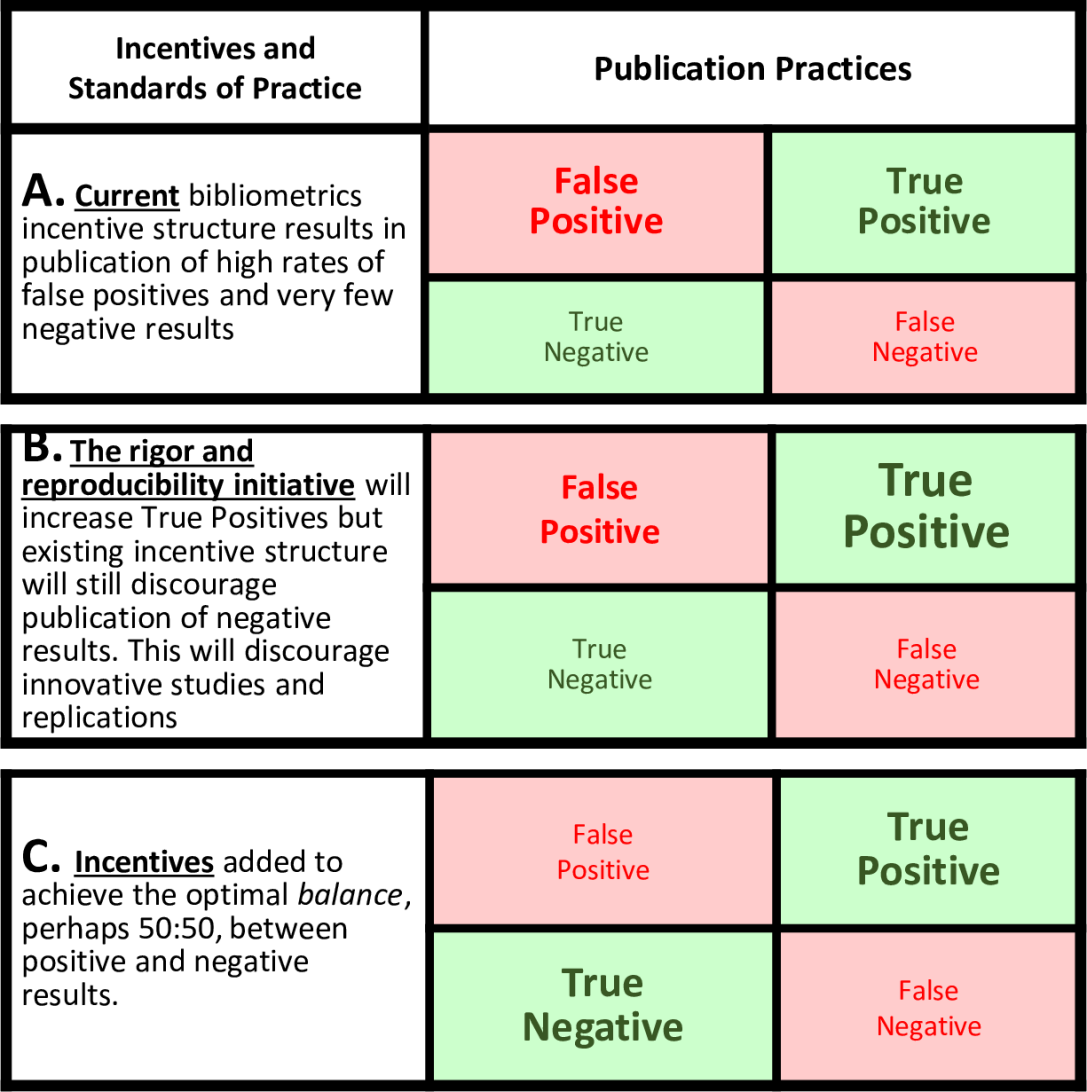
Standards of practice and publication patterns. Font size represents relative numbers of publications in each category.

The pressure to publish lots of highly-cited discoveries affects the perceptions, judgments and decision-making processes of scientists. While the effects of that pressure are largely outside conscious awareness, it encourages the use of exploratory procedures and discourages the use of rigorous confirmatory procedures that limit the effects of bias and filter out false positives [9]. High quality procedures that control for the effects of experimenter bias substantially reduce the number of more highly-cited new, statistically significant results and increase the number of negative results [10–14]. An emphasis on low-quality exploratory methods have thus evolved as the standard of practice because they are adaptive for individual scientists, given the current system of incentives [15–20]. The consequence is that many invalid and nonreproducible findings are produced and published and not efficiently eliminated with rigorous replication studies which results in the publication of large numbers of nonreproducible false positives [21], leading to what is now recognized as a “reproducibility crisis”. For example, Begley reported that 89% of highly cited landmark preclinical studies of novel targets and interventions for cancer were not reproducible [22] (Fig 2A).

Effective procedures are available to increase the validity and reproducibility of the results of studies, such as: randomization, allocation concealment, blinding, using power analyses to ensure adequate sample size, pre-specifying primary outcome measures and analytical plans and avoiding extensive post-hoc analyses [9]. The National Institutes of Health (NIH) is developing policies to address problems with rigor and reproducibility in NIH-funded research (NOT-OD-16-011, NOT-OD-15-015, NOT-OD-16-149), and many of the major professional societies and publishers have endorsed guidelines to address these issues (Endorsing Associations, Journals, and Societies). These changes will likely increase the reproducibility of studies by decreasing the proportion of false positives and false negatives and increasing the proportion of true positives reported in the literature (Fig 2B). However, so long as the incentives continue to emphasize and reward only statistically significant discoveries and highly-cited papers, there will be resistance to accepting the use of rigorous confirmatory methods and publication of the results regardless of the outcome.

To change current standards of practice it is important to develop incentives for the individual scientist that are consistent with the interests of the scientific community and society as a whole [23]. It has been suggested that scientific progress is maximized if the expected results are achieved only approximately 50% of the time [24, 25], but negative results are disappearing from the literature and now make up only 10% or less of current publications [16]. Phase II-IV clinical trials are conducted using more rigorous methods than nonclinical studies [9, 26], clinical trials consistently include replication studies as a regular part of the clinical approval process, and they produce a much higher proportion of valid but negative results than nonclinical studies [9]. Therefore, we examined a sample of randomized, controlled, Phase II-IV clinical trials registered at ClinicalTrials.gov to determine what metrics and incentives could be developed to encourage scientists to use high-quality methods, to conduct confirmation/replication studies, to publish the results even if they are not statistically significant, and to achieve an appropriate balance between positive and negative results (Fig 2C).

For example, if the publication of negative results leads to a decrease in the activity and resources devoted to that topic, thus diverting resources away from topics that lack promise, decreases in research activity might be used as a new metric and incentive to encourage scientists to use high-quality methods, to conduct rigorous confirmation/replication studies, and to publish the results even if they are not statistically significant. We used the number of papers being published on a topic as a measure of research activity. The number of publications has been used as a measure of the overall level of research activity being devoted to specific areas of investigation for more than 50 years, and this measure is still in common use [27–31].

## Methods

This exploratory study analyzed a dataset produced and reported previously by co-authors NK and KT [32]. The basic procedures will be summarized here but for more detailed descriptions of the original search procedures used to identify the clinical trials and their related publications see Khan et al. [32]. ClinicalTrials.gov was searched on January 4, 2012 using the term “rheumatoid arthritis” and a first received date of December 31, 2011 or earlier. Trials were excluded based on the following characteristics: condition other than rheumatoid arthritis, Phase I or observational study types, nonrandom subject allocation, recruitment status terminated or active, primary outcome of safety or completion date after December 31, 2009 (to provide a minimum of 2 years from study completion to initial publication search). The “primary completion date” (defined at ClinicalTrials.gov as the date of collection of primary outcome measure on the last patient to be enrolled) or, when the primary completion date was unreported, the study completion date defined by the trial’s investigators was used to determine the study completion date.

In January 2012 peer-reviewed publications were searched for study trials using a standardized strategy. First, the ClinicalTrials.gov record was reviewed for links to publications resulting from the registered trial. Next, using PubMed, Medline was searched sequentially using NCT ID number and the terms “rheumatoid arthritis” and “study intervention(s)”. The search results were refined, if needed, by specifying study design features, the name of the principal investigator, and the primary outcome. The Google Scholar database was similarly searched if the Medline search was unsuccessful. Trial publication was confirmed by matching study characteristics at ClinicalTrials.gov with the description in the manuscript. The article reporting primary outcome results was chosen if multiple publications originated from a single study. Principal investigators or study sponsors of trials with an unsuccessful publication search were contacted by email (up to 3 emails) to inquire about publication status.

Outcomes (i.e., positive, statistically significant results or negative, non-statistically significant results) were determined by examining the results of the primary outcomes reported in the published manuscripts. If the primary outcome was unspecified, then the outcome used for sample size calculation or first reported clinical efficacy outcome was used. The study outcome was considered positive if any experimental intervention arm had a statistically significant result for the primary outcome, unless safety concerns about the experimental intervention necessitated the authors not to recommend the intervention [32]. For unpublished studies, outcomes were determined from ClinicalTrials.gov, Thomson Reuters’ Web of Science (http://ipscience.thomsonreuters.com/product/web-of-science/), online archives of abstracts presented at annual meetings of the American College of Rheumatology (2006–2011), the European League Against Rheumatism (2002–2011), industry-sponsored web sites, and Google searches. If the experimental intervention was not significantly different from the control group with respect to the primary outcome, the result was classified as negative. Because many of the studies included active controls, a negative outcome simply meant that the experimental intervention was not significantly better than the active control.

All searches were updated and finalized by July 7, 2012. The resulting dataset included 143 Phase II-IV clinical trials focused on interventions for a single clinical indication, rheumatoid arthritis, registered at ClinicalTrials.gov and completed by December 31, 2009 [32]. Additional studies were excluded from the present analyses because the outcomes were unknown (n = 15), the results were published after 2012 (n = 8), they involved complex interventions and/or strategy development and it was not possible to determine appropriate search terms (n = 8), and because they were not published in English (n = 2). All inclusion and exclusion decisions were made before proceeding with the data collection and analyses conducted in the present study (S1 Fig). The remaining dataset included 110 Phase II-IV clinical trials focused on interventions for a single clinical indication, rheumatoid arthritis: 100% included placebo or active controls, 100% included randomized subject allocation, and 92% were blinded (86% were double-blind and 6% were single-blind).

For each study included in the present analysis, search terms were identified to determine the level of activity of research on that topic for each year during a five-year period, as measured by the number of other publications on the same topic as the study included in our analysis. Relevant search terms were identified for each intervention being tested, including the name of the molecular target, the experimental drug and all other drugs for the same molecular target. For nonpharmacological interventions, search terms were identified from clinicaltrials.gov records and publications. All these terms were combined using the Boolean operator ‘OR’, and these terms were then combined with the search term “Rheumatoid Arthritis” using the Boolean operator ‘AND’.

Level of research activity was calculated from 2 years before until 2 years after the year of publication of each study included in our analysis. Therefore, for a clinical trial examining the efficacy of a tumor necrosis factor (TNF) inhibitor for rheumatoid arthritis, the activity level of that topic was represented by the number of other papers published each year, from 2 years before until 2 years after the year of publication, with terms relevant to tumor necrosis factor and rheumatoid arthritis in the title, abstract or keywords. For unpublished studies, the year the study was completed, as shown in ClinicalTrials.gov was substituted for year of publication. Searches were conducted in Scopus and Thomson Reuters’ Web of Science. As the number of publications on the different topics was very consistent between Scopus and Web of Science, r(110) = 0.99; the publication numbers from Scopus were used in these analyses.

In addition, the number of citations in Scopus within two years of publication was included in the analysis for each of the clinical trials in the sample. Citations over the initial two-year period from the time of publication is a standard bibliometric measure of productivity. Transformations of the raw citation numbers were also included in the analyses using sophisticated algorithms to normalize for year of publication and field of study: the Relative Citation Ratio (RCR) developed at NIH, computed and made available to the public using the tool iCite (https://icite.od.nih.gov/) [33]. Because the RCR is normalized for year of publication and field of study, it has been suggested as an alternative to raw citation numbers that would be free from some of the problems related to the use of raw citation counts.

We make all the data included in our analyses available to the scientific community for further study (S1 Dataset). The supplementary data file includes the clinicaltrials.gov study number (column heading “NCT ID”), the PubMed publication numbers (column heading “PubMed ID”), search terms (column “Scopus search terms for searches conducted from October to December 2015”), study outcomes (column heading “Study Outcome Positive or Negative), interventions (column heading “Intervention”), publication status (column heading “Publication Status”), the number of publications found with the Scopus search terms from two years before until two years after the publication of the trial publications (heading “Research Activity Levels From 2 years Before until 2 years After Trial Publications”, activity levels for each of those five years are in columns I through M), the number of citations for the trial publications (column heading 2-year citation number), and the Relative Citation Ratio or RCR (column heading “RCR (from iCite—at https://icite.od.nih.gov/analysis on June 4, 2016)”).

All analyses were conducted on the data included in the supplementary data file using SAS 9.4.

## Results

**Fig 3:**
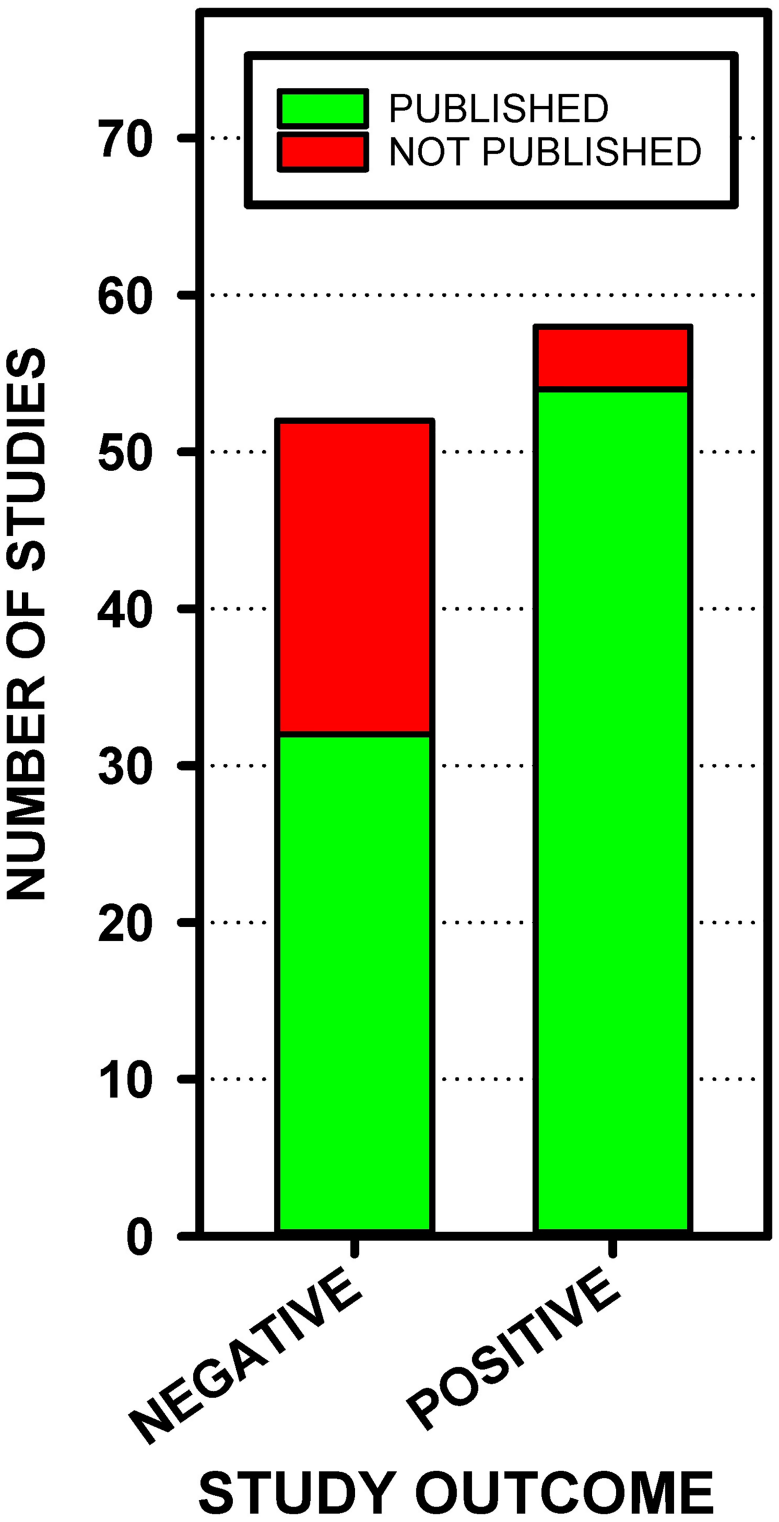
Number of studies published and not published with positive or negative outcomes.

The 110 Phase II-IV clinical trials included in our analysis produced a large proportion of studies that failed to confirm the hypothesis and resulted in negative, non-statistically significant results: 52 (47%) had negative outcomes, and 58 (53%) had positive outcomes (Fig 3). Assuming the unpublished studies with unknown outcomes were distributed the same as the unpublished studies with known outcomes, it is estimated that 52% of all the studies conducted produced negative, non-statistically significant effects. Studies that successfully confirmed the hypotheses and resulted in positive, statistically significant outcomes were more likely to be published. Of the studies with positive outcomes, 93% were published, while only 62% of the studies reporting negative results were published. *χ*^2^ analyses showed that the proportion of nonpublished studies was significantly higher for trials with negative results than trials with positive results, *χ*^2^(1, N = 110) = 16.01, *p* < 0.0001 (Fig 3).

**Fig 4:**
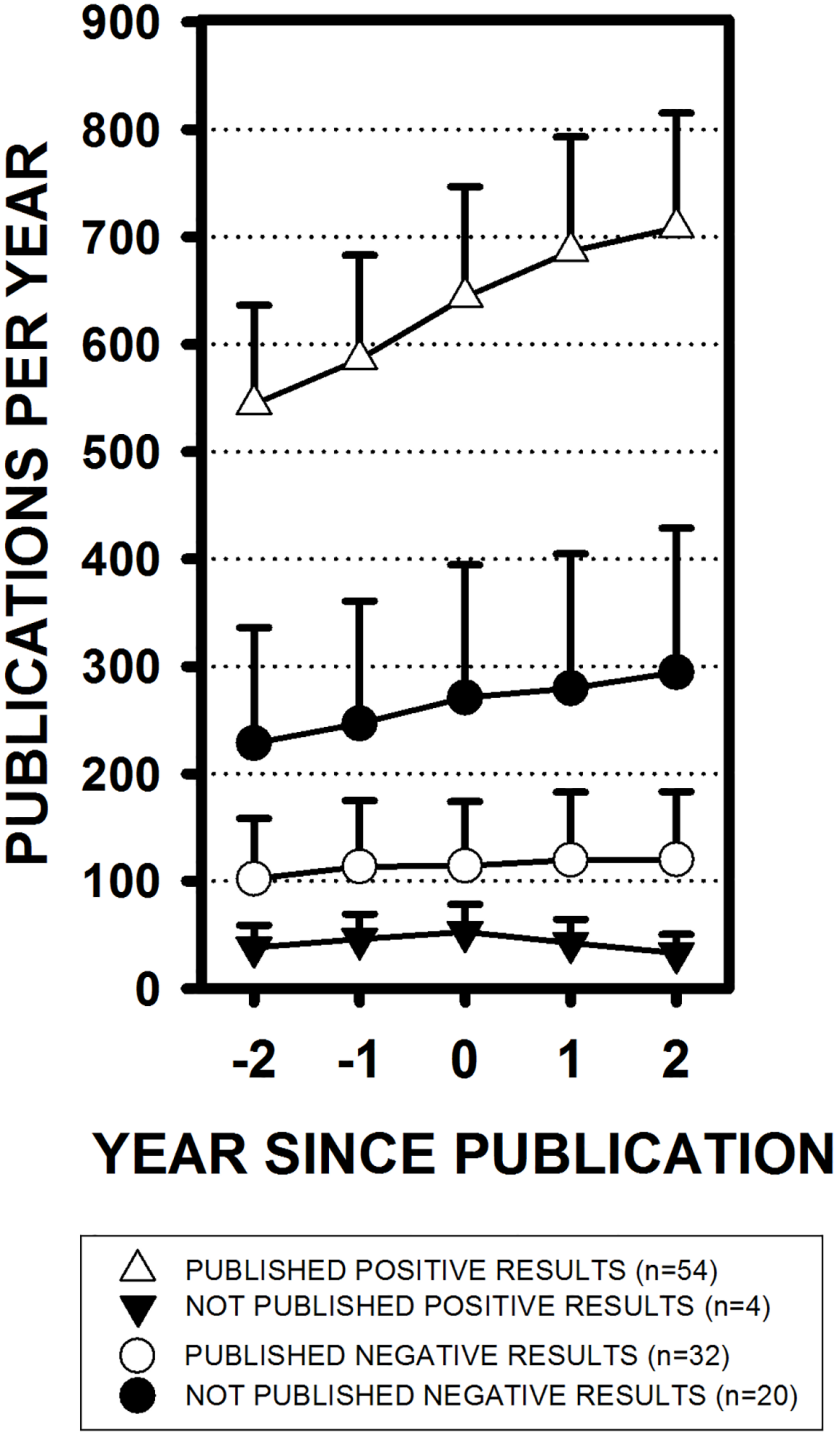
Research activity levels by study outcome. The number of papers published each year on the same topics as the studies included in the present analysis (means ± SEM). Publication years range from two years before (-2) until two years after the year the studies included in the present analysis were either completed or published (year 0). For example, even two years before the publication of the studies reporting positive results, on average, 544 ± 92 papers were already being published each year on those same topics. Two years before the publication of the studies reporting negative results, only 102 ± 56 papers were being published each year on those topics.

Overall, studies reporting negative outcomes did not produce significant decreases in research activity, as measured by the number of other publications on those same topics. However, that was primarily due to a floor effect. A between-groups repeated measures ANOVA was conducted with year from publication as the repeated measure and a planned contrast between the publications reporting positive results and the publications reporting negative results. In general, publications reporting positive results were focused on topics that were already being much more actively investigated than publications reporting negative results, *F*(1, 106) = 15.04, *p* = 0.0002 (Fig 4). Only a few studies with positive outcomes were not published (n = 4), and those studies were conducted on topics with very low levels of research activity.

Based on our search terms, there were large differences in the levels of research activity between different topics or interventions. For example, more than 1,000 papers were being published each year related to tumor necrosis factor and rheumatoid arthritis; but, less than 10 papers were being published each year related to A3 adenosine receptors and rheumatoid arthritis. The 110 studies with known outcomes were divided into five groups (n’s = 22) based on research activity levels in the same year that the studies were published (Table 1). For example, group 1 includes studies with research activity levels of 0 to 17, with a median of 7.

**Table 1:** Studies divided into 5 groups based on research activity levels.

Group	Number of Studies	Range of Activity Levels in Each Group			Total Number of Positive & Negative Studies		Number of Published Studies	
		Minimum	Median	Maximum	Positive	Negative	Positive	Negative
1	22	0	7.0	17	7	15	5	7
2	22	18	30.5	57	9	13	9	11
3	22	58	88.0	128	9	13	7	11
4	22	130	265.0	504	14	8	14	2
5	22	538	1829.0	2058	19	3	19	1

**Fig 5:**
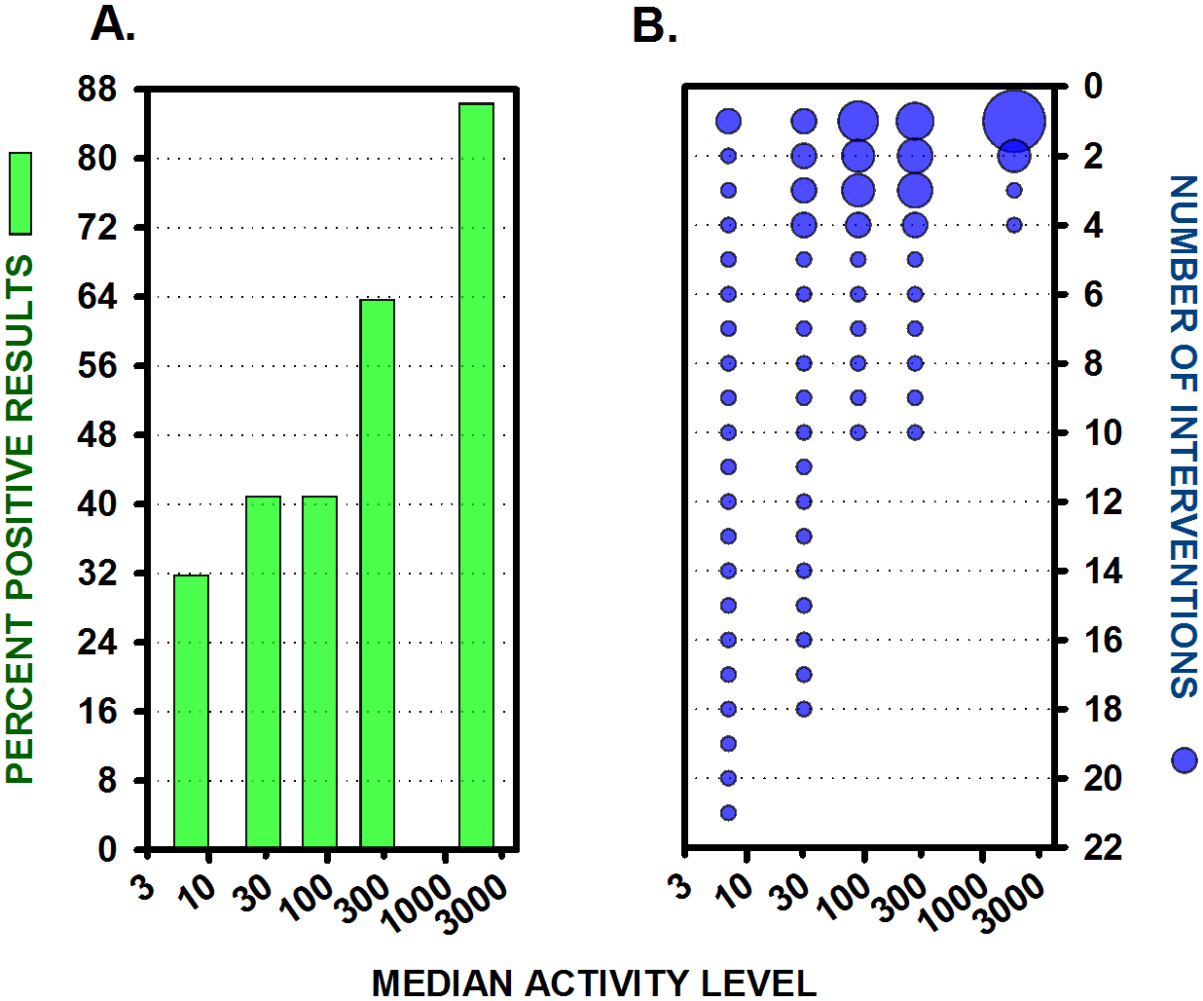
Success rates increase and number of topics decrease as research activity levels increase. (A) Percent successful, or positive, statistically significant results, at median activity levels for 5 equal groups (n’s = 22). (B) The number of interventions or topics of research is shown at each activity level. Symbol size indicates the number of studies being conducted on the same topic. The Y axes is inverted in this panel in order to convey the concept that, over time, only a small number of reliable findings rise to the top.

For studies on topics with the lowest activity levels, a median of seven other papers were published on those same topics in the same year, and success rates (the percentage of positive outcomes) increased as activity levels increased: from 33% positive outcomes at the lowest activity level with only seven other publications per year; to more than 86% positive outcomes at the highest activity level with almost 2,000 other publications per year on the same topic. A χ2 analysis showed that the proportion of positive results was significantly different across the 5 groups with different activity levels: *χ*^2^(4, N = 110) = 17.36, *p* = 0.0016 (Fig 5A).

In addition, differences in activity levels were related to the range and diversity of topics being examined. As the levels of research activity increased, the number of different topics being examined decreased, from many topics or interventions each being examined in one or two studies at the lowest activity levels, to very few well-established topics, each being examined in numerous studies at the highest activity levels. A χ^2^ analysis showed that the number of different topics tested was significantly different across the 5 groups with different activity levels, *χ*^2^(4, N = 110) = 34.77, *p* < 0.0001. For example, the 22 studies at the lowest research activity level examined 21 different topics, but the studies on topics with the highest research activity levels were concentrated on only 4 different topics: 17 of the 22 studies at that highest level of activity were focused on tumor necrosis factor and rheumatoid arthritis (Fig 5B).

**Fig 6:**
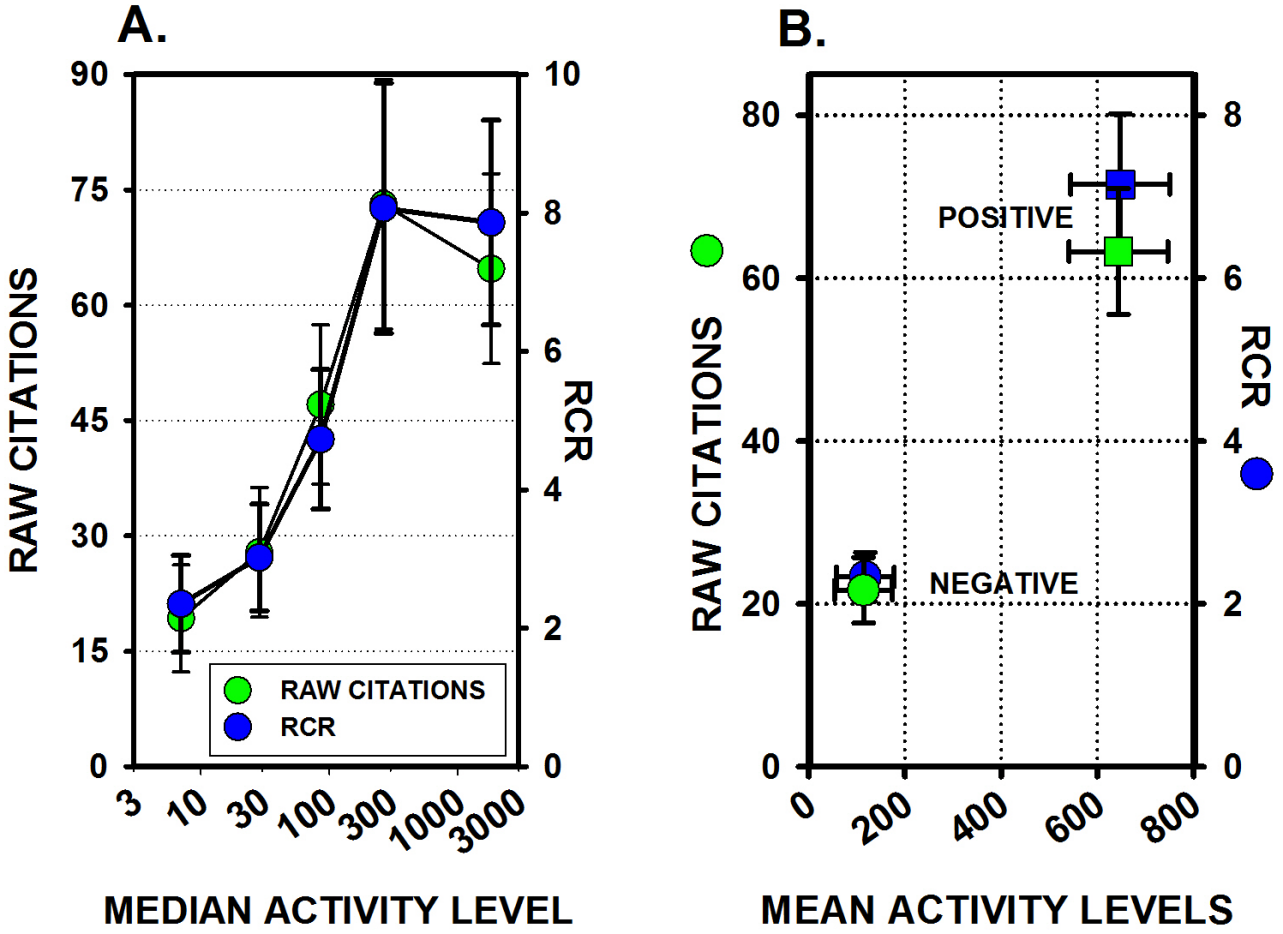
Raw citation counts and RCRs are related to research activity levels. (A) RCR values were calculated approximately 8 years after the papers were published but RCR values and 2-year raw citation numbers were very highly correlated, r(86) = 0.96, and both raw citation numbers and RCR values increased as activity levels increase. (B) Publications reporting positive, statistically significant results were more highly cited than studies reporting negative, non-statistically significant results, and those differences were still evident even with article-level normalized RCR values.

The levels of research activity were also related to citation numbers. There was a wide range of citation rates at every level of activity, but as activity levels increased, the range and the means of the citation numbers increased. A between-groups ANOVA showed statistically significant differences in 2-year citation numbers across the 5 groups with different activity levels, *F*(4,81) = 3.57, *p* = 0.0099 (Fig 6A). Article-level citation numbers normalized for year of publication and field of study, the Relative Citation Ratios (RCR), also increased as activity levels increased, *F*(4,81) = 4.20, *p* = 0.004 (Fig 6A).

Negative results were more frequent at lower activity levels where citation numbers were lower, and positive results were more frequent at higher activity levels where citation numbers were much higher (Fig 6B). In addition, publications reporting positive, statistically significant results were cited much more often than publications reporting negative results: 63.26 ± 7.74 citations for positive studies (mean ± SEM), and only 21.6 ± 4.02 citations for negative studies. A between-groups ANOVA showed statistically significant differences in 2-year citation numbers from publications reporting positive results compared to publications reporting negatives results, *F*(1, 84) = 13.98, *p* < 0.0003 (Fig 6B). Differences between positive and negative publications were still just as clearly evident even if raw citation numbers were normalized for year of publication and field of study using the RCR, *F*(1,84) = 17.53, *p* < 0.0001 (Fig 6B).

## Discussion

Intense selection pressure for employment and career advancement [7] combined with an incentive system that rewards the publication of large numbers of highly-cited discoveries leads to a large proportion of papers that are not reproducible [21, 22, 34]. Efforts are underway to address this reproducibility crisis by making high quality methods the standard of practice. However, so long as scientists are selected and rewarded for their ability to publish large numbers of statistically significant, highly-cited papers, there will be resistance to efforts to increase rigor and reproducibility.

Nonclinical studies rarely include rigorous procedures such as blinding and randomization [10, 11, 22, 35–42], and negative results account for only 10% of the papers being published [16]. The present study examined a set of Phase II-IV clinical trials registered at ClinicalTrials.gov to determine if metrics and incentives could be developed to encourage scientists to use high-quality, confirmatory methods and to publish their results even if they are not statistically significant. Of the trials included in the current analysis, 100% included placebo or active controls, 100% included randomized subject allocation, 92% were blinded, and approximately 50% produced negative results. Initial examination of this sample of Phase II-IV clinical trials revealed a pattern consistent with what has already been reported in the literature: [1] studies using high-quality methods to ensure the validity of the results often produce negative, non-statistically significant results that fail to support the hypothesis [10–12], [2] publications of positive, statistically significant effects are cited much more often than studies reporting negative, non-statistically significant effects [13, 14], and [3] partly because they are not highly valued, non-statistically significant results are often not published [43].

Studies reporting negative outcomes did not produce significant decreases in research activity, as measured by the number of other publications on those same topics. However, that was primarily due to a floor effect. Most of the negative results were produced in studies testing a wide range of different topics that were not yet being very actively investigated. Initial studies that detected a significant effect attracted more resources and increased research activity, but with mixed success, more negative results and eliminations, leading to only a few well-established topics surviving with very high activity levels that consistently and reliably produced the expected, positive, statistically significant effects.

These findings are consistent with the way scientific knowledge is expected to increase in well-controlled clinical trials where rigorous procedures and strict replication/confirmation studies are accepted as the standard of practice. Initial studies of new and perhaps more innovative topics and approaches that are not yet being actively explored have higher levels of uncertainty and higher failure rates. As more studies are conducted and more information is collected, our knowledge increases, uncertainty decreases, and results become more predictable. In general, initial, high quality studies of novel subjects produce the largest amount of information relative to what is already known, and less information is gained with each successive study. Innovative, rigorous studies of new topics that support the hypotheses only 30–60% of the time provide more information than studies of well-established topics that reliably produce the expected, positive results more than 90% of the time [44–46].

Thus, the present study suggests that the number and diversity of topics being investigated, success rates, research activity levels and the amount of information produced might all be useful as measures to manage innovation and risk, and as incentives to encourage scientists to conduct and publish rigorous innovative studies and replications, even if the results are not statistically significant. In contrast, the current bibliometric incentives discourage rigorous procedures, strict replication/confirmation studies and publication of negative, nonstatistically significant results. Furthermore, the present results suggest that, to the extent that higher quality methods are accepted as the standard of practice, the current bibliometric incentives will discourage innovative studies and will put pressure on scientists to shift their research to areas of study that are already being more actively investigated. Studies of topics that are already being very actively investigated are generally less innovative and informative, but they are more likely to produce positive, statistically significant results and higher citation rates. This is consistent with what others have reported [47].

It is also important to note that even sophisticated transformations of the current bibliometric measures, including article-level citation values normalized for year of publication and field of study, such as the RCR, do not address this issue. The RCR has been described as a measure of ‘influence’ that addresses some of the problems related to the use of raw citation counts [33, 48], but it is not clear that the RCR is fundamentally different than raw citation counts. In the present study, the RCR was highly correlated with raw citation counts, and scientists select other papers to cite based primarily on their rhetorical utility, to persuade their readers of the value and integrity of their own work. Papers are not selected for citation primarily based on their relevance or validity [49, 50]. Even the father of the Science Citation Index (SCI), Eugene Garfield, noted that citations reflect the ‘utility’ of the source, not their scientific elegance, quality or impact [51]. Authors cite only a small fraction of relevant sources [13, 52], and studies reporting robust, statistically significant results that support the author’s agenda have greater utility and are cited much more often than equally relevant studies that report small or non-statistically-significant effects [13, 14, 52–56]. As Peter Moore at Yale University has noted, “If citations are what you want, devising a method that makes it possible for people to do the experiments they want … will get you a lot further than …discovering the secret of the Universe” [57].

The RCR is also very new and has not yet been subjected to extensive independent evaluation. It’s validation is primarily based on correlations between the RCR and evaluations of the content of the publications by expert reviewers [33]. However, in an independent analysis, Bornmann and Haunschild reported a correlation of only 0.29 between the RCR and expert reviewer evaluations, which means that less than 10% of the variance in expert evaluations is accounted for by the RCR [58]. Janssens also reported that 80% of the citations in the co-citation network used to calculate the RCR are cited only once, so the RCR calculation may be based largely on co-citations that have little relevance [59]. Furthermore, the developers of the RCR acknowledge that the RCR may not be reliable in papers published within the last 2–3 years or cited only 5 times or fewer [60]. More than 50% of all publications have been cited only 5 times or fewer which suggests that most RCR values may not be reliable.

To maximize scientific progress and productivity it is important to develop metrics and incentives that align the interests of the individual scientist with the interests of the scientific community and society as a whole [20, 23]. This study shows that more appropriate metrics and incentives can be developed, but of course more work is needed to develop and validate alternatives to the current bibliometric incentives. For example, the present study focused on clinical trials for rheumatoid arthritis, so it will also be important to determine how to specify topics and measure research activity levels for topics in broader, less constrained areas of nonclinical research. In addition, it will be important to evaluate ‘positive’ and ‘negative’ results in nonclinical publications which often include multiple experiments and numerous outcome measures.

The optimal range and overall balance of positive and negative results will also need to be examined. It has been suggested that scientific progress is maximized if overall success rates are approximately 50% [24, 25]. Rigorous FDA-regulated confirmatory trials of new therapeutics cut the number of therapeutics advancing to the next stage by about 50% each round, resulting in only about 10% surviving to be approved as safe and effective after only a few iterations of confirmatory tests [61]. More innovative therapeutics have higher failure rates. For example, only 6% of new molecular entities are eventually approved for clinical use, while 22% of treatments that are only minor variations of already-approved molecular structures (me-too drugs) survive the filtering process to be approved [61]. The outcomes of the Phase II-IV clinical trials examined in the present analysis were balanced approximately 50:50 between positive and negative results but exactly how nonclinical studies should be distributed across the continuum from new and highly innovative topics at one end, to the few remaining well-established topics supported by multiple replication/confirmation studies, remains to be determined.

The present results also suggest that it may still be possible to measure decreases in research activity levels, but future efforts will need to focus on topics with fairly high levels of research activity so that decreases can be detected. The number and proportion of negative results will also need to be evaluated for each topic, and the duration of study will need to be extended. Two years is not adequate to detect significant decreases in research activity levels because it often takes several years for numerous negative results to accumulate, and several more years before projects that are already in the pipeline have published their results.

To be clear, bibliometric measures are not necessarily dangerous or inappropriate, they are only problematic when they are overemphasized in decision-making processes. Bibliometric measures, including the alternative measures we are proposing, should only be used to augment, never substitute for or replace expert judgment [62–64]. Our results suggest that the overall pattern of a range of different parameters should be considered. For example, determining the quality of the methods and the likelihood that the results will be reproducible must be determined by careful, expert evaluations of the experimental methods [65]. It may also be assumed that topics that are not being very actively investigated are exploring ideas that are novel and more innovative, but it is possible that such topics were already investigated in the past and did not prove fruitful. Expert evaluation is required to determine if studies are exploring significant and meaningful issues, and measures of research activity might also be better estimated by experts who understand how much relevant research has been conducted, including all previous published and unpublished studies. In addition, while most highly cited papers in areas that are already being very well explored are reporting only limited, incremental increases in knowledge, some of them are reporting revolutionary, paradigm-shifting results, and expert evaluations are required to make those discriminations as well [57]. And of course, assessing study outcomes and determining what the optimal distribution of success rates might be are dependent on expert evaluations of the content of the publications.

Previous theoretical and mathematical models have suggested that scientific progress and productivity is dependent on the exploration of diverse, innovative hypotheses to advance the frontiers of knowledge, combined with critical replication/confirmation studies to eliminate results that are not reproducible [1–5, 20, 44, 66]. If rigorous, innovative studies of significant issues and publication of valid, reproducible results are desired, the best way to achieve those objectives is to explicitly evaluate and reward scientists based on those criteria. The present results suggest that metrics and incentives can be developed to reward scientists to achieve those objectives. Such a system of incentives should produce a portfolio of projects and publications that range from a large number of different, new and innovative ideas and hypotheses with high failure rates, down to a small number of well-established subjects that reliably produce the expected results. This will increase scientific productivity and the integrity of the scientific literature and result in some highly-cited papers, but it will also reduce publication rates and produce a significant proportion of poorly cited publications reporting valid negative results.

## Supporting information

S1 Fig Flow chart of trial. Flow chart of selection of clinical trials (CTs) of treatment of rheumatoid arthritis (RA) registered at ClinicalTrials.gov.
(TIF)

S1 Dataset Spreadsheet of all raw data. Spreadsheet of clinicaltrials.gov study number, search terms, study outcome (positive or negative), and indication that results were published or not published.
(XLSX)
