# Supplementary figures and images for: Scientific productivity: An exploratory study of metrics and incentives
Source: PLoS One. 2018 Apr 3;13(4):e0195321. doi: 10.1371/journal.pone.0195321 (PMC5882165; doi:10.1371/journal.pone.0195321)

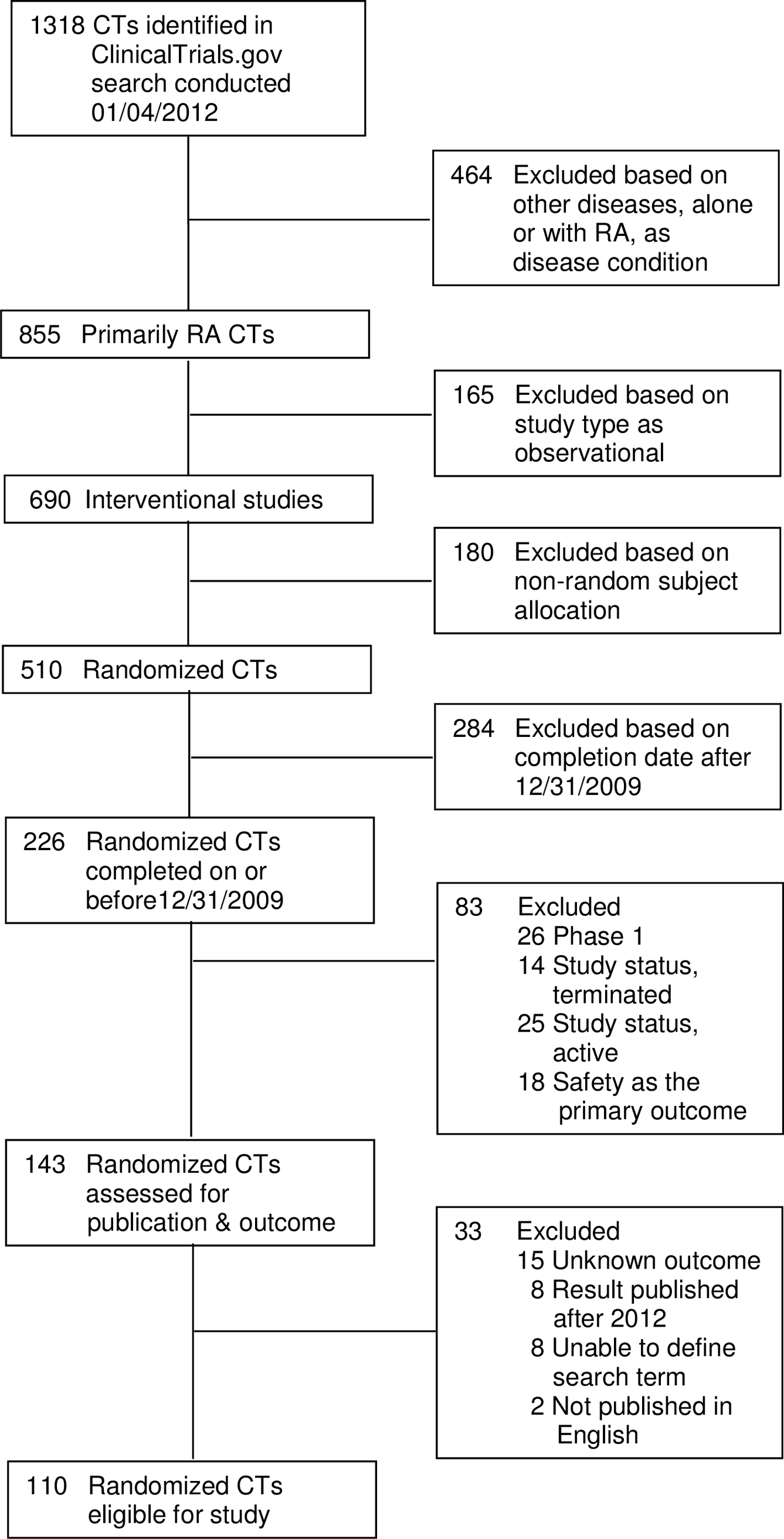

Supplement: S1 Fig — Flow chart of selection of clinical trials (CTs) of treatment of rheumatoid arthritis (RA) registered at ClinicalTrials.gov. (TIF) [file pone.0195321.s001.tif]
